# Concordance of Child Self-Reported and Parent Proxy-Reported Posttraumatic Growth in Childhood Cancer Survivors

**DOI:** 10.3390/cancers13164230

**Published:** 2021-08-23

**Authors:** Veronika Koutná, Marek Blatný, Martin Jelínek

**Affiliations:** Institute of Psychology, Czech Academy of Sciences, 602 00 Brno, Czech Republic; blatny@psu.cas.cz (M.B.); jelinek@psu.cas.cz (M.J.)

**Keywords:** posttraumatic growth, benefit finding, childhood cancer survivors, parent–child concordance

## Abstract

**Simple Summary:**

In pediatric cancer settings, parents can be asked to provide information about the impact of cancer on the child. However, their assessment of the child may not be accurate. Research has shown that parents tend to underestimate the quality of life of their child following pediatric cancer. Little is known about the accuracy of parental reports of posttraumatic growth (PTG) as a consequence of pediatric cancer. Our study aimed to examine concordance of parent- and child-reported PTG with taking into account the parents’ own level of PTG. We found poor parent–child concordance, with parents reporting higher levels of PTG for their children than the children themselves. When assessing their child’s PTG, parents are influenced by their own level of PTG. These findings provide implications for the research on psychosocial outcomes of pediatric cancer using a multi-informant perspective as well as for the topic of veracity of PTG in general.

**Abstract:**

This article aimed to analyze concordance of parent- and child-reported child posttraumatic growth (PTG) following pediatric cancer, the influence of the parents’ own level of PTG on the level of concordance and the influence of the parents’ and the child’s own level of PTG on the parents’ proxy reports of PTG in the child. The sample included 127 parent–child dyads. The children provided self-reports of PTG and the parents provided reports of their own as well as the child’s PTG. Overall, the results showed poor parent–child agreement on the child PTG, with the parents proxy-reporting higher levels of PTG than the children. The parents’ proxy reports of the child PTG were the most accurate at the lowest levels of the parents’ own level of PTG. The parents’ own level of PTG was a stronger predictor of the parents’ proxy reports than the child self-reported PTG. The results suggest that parents are not very accurate reporters of PTG in the child; therefore, their reports should be completed with child self-reports whenever possible.

## 1. Introduction

Pediatric cancer has been widely studied in relation to posttraumatic stress symptoms (PTS) in childhood cancer survivors as well as in their parents. The results of these studies have shown that survivors, in general, are not at increased risk of severe PTS or full posttraumatic stress disorder compared to the normal/healthy population [1,2]. Parents (especially mothers) report higher levels of PTS than childhood cancer survivors themselves [3]. Higher levels of PTS in mothers of pediatric oncology patients are associated with higher levels of PTS in the child [4]. In addition to negative psychosocial consequences such as PTS, both childhood cancer survivors and their parents report posttraumatic growth (PTG) defined as positive changes following traumatic experience, typically in the domains of relating to others, new possibilities, personal strength, spiritual change and appreciation of life [5]. The prevalence of PTG in survivors and their parents is reported to be between 80–90% [6].

Although research in PTS in pediatric oncology suggests an association between parent and child adaptation, the studies focused on the parent–child associations in PTG following pediatric cancer are very limited and their findings are mixed. Michel et al. [7] reported no parent–child association in perceiving positive changes following pediatric cancer. On the other hand, Yaskowich [8] reported a parent–child association in PTG, with higher levels of PTG in the children who are aware of PTG in their parents. Several other studies focused on PTG in childhood cancer survivors and their parents simultaneously, but only Michel et al. [7] and Yaskowich [8] analyzed the parent–child connections in PTG directly. However, some studies provided indirect support for the parent–child associations in PTG by pointing to the role of the parent–child relationship and factors of parenting in child PTG following pediatric cancer [9,10,11].

Parents may shape the process and outcomes of child psychosocial adaptation, including both PTS and PTG, through the socialization of coping and emotions (for review, see Koutná and Blatný [12]). They serve as the key sources of social support for childhood cancer survivors [13] and, simultaneously, they can be used as sources of information when the child is unwilling or unable to provide relevant information on their own (due to health complications or insufficient cognitive maturity).

### 1.1. Parents as Proxy Reporters

Although Varni et al. [14] argued that even young children can reliably self-report health-related quality of life using an age-appropriate method, a multi-informant approach including information from the child as well as other sources (e.g., parents, teachers, physicians) is recommended for a comprehensive assessment of the child’s well-being [15]. In studies using a multi-informant perspective, parents are asked to provide reports concurrently with the child. Parents are undoubtedly an important source of information about their children but research on the quality of life has shown that the perspectives of children and parents may be different. Parents tend to underestimate the quality of life of their child following pediatric cancer treatment [16]. The agreement between the child’s self-report and the parents’ proxy report of the child’s quality of life is higher for more easily observable domains (e.g., physical functioning) than for less observable ones (e.g., emotional domain) [15].

Tomlinson et al. [17] identified several psychological factors related to discrepancies in child’s self-reports and parents’ proxy reports including projection of the parents’ own feelings and assumptions. Similarly, Smith et al. [18] included parental affect, expectations and beliefs among the psychosocial factors affecting the parents’ proxy reports of the child’s symptoms. However, the concordance of the self-reports and the parents’ proxy reports of health-related quality of life of childhood cancer survivors appears to be better than in healthy parent–child dyads [19]. Good agreement between the child’s self-reports and the parents’ proxy reports was also found in the research of PTS in childhood cancer survivors and even in the parents with high own levels of PTS [20,21].

### 1.2. Corroborating Self-Reports of PTG

The field of PTG research offers one slightly different perspective on using parents (significant others) as proxy reporters. The majority of PTG studies face criticism of over-reliance on self-report measures, and PTG research in pediatric oncology is no exception. The veracity of PTG reports obtained by self-report measures is often questioned because it does not allow distinguishing between actual positive changes and other concepts such as positive illusions. To address this issue, some authors suggested corroboration of self-reports by reports of others [22]. Therefore, the parents’ proxy reports of PTG in their child could serve as a means of verifying the child’s self-report. This approach was utilized in several studies focused on adults and their spouses or other significant others.

For example, Shakespeare-Finch and Enders [23] found a significant correlation (*r* = 0.69) between the PTG self-reported by adult trauma survivors and proxy reports of their significant others (spouses, family members and close friends). Blackie et al. [24] reported corroboration of PTG in adults by proxy reports of others. Weiss [25] found a significant correlation (*r* = 0.51) between the PTG score self-reported by breast cancer survivors and the PTG score proxy-reported by their spouses. Moore et al. [26] analyzed agreement in the PTG self-reported by adult cancer patients and their caregivers. They found a significant correlation (*r* = 0.47) between the total PTG scores self-reported by patients and the ones proxy-reported by caregivers. On the other hand, Helgeson [27] found little corroboration of positive changes reported by breast cancer survivors and their significant others.

To the best of our knowledge, the agreement between the child’s self-report and the parents’ proxy report of positive outcomes resulting from pediatric cancer has not been widely studied, at least not with the appropriate attention devoted to the parents’ own PTG as well. The only study assessing and analyzing all three sources of information about PTG is the dissertation thesis by Yaskowich [8], which did not find a significant difference between the child’s self-report and the parents’ proxy report of PTG. Our study aim to analyze (1) the level of concordance of parent- and child-reported child PTG following pediatric cancer, (2) the influence of the parents’ own PTG on the level of the parent–child concordance and (3) the influence of the parents’ and the child’s own PTG on the parents’ proxy report of PTG in the child.

## 2. Materials and Methods

The sample of this study is a part of a larger sample recruited for the QOLOP (Quality of Life Longitudinal Study in Pediatric Oncology Patients) project launched in 2006. This project aims to longitudinally assess the quality of life in childhood cancer survivors (2–5 years off-treatment at the beginning of the project). The project was approved by the ethics committee of the participating hospital. All the respondents were thoroughly familiar with the aims of the project and signed an informed consent form to participate in the study.

### 2.1. Child Self-Reported and Parent Proxy-Reported PTG

PTG in the child was assessed using the Benefit Finding Scale for Children (BFSC; [7,28]). BFSC is a widely used 10-item measure asking about the degree of positive changes following a trauma on a 5-point scale. It was administered to childhood cancer survivors, who reported their own positive changes following cancer survival (child self-reported PTG), and to their parents, who reported positive changes in their child (parent proxy-reported PTG). For example, the children were asked to agree/disagree with the following: “*Having had my illness has helped me become a stronger person*”. The same item for parents was formulated as “*My child’s illness has helped him/her become a stronger person*”. The reliability of the BFSC was good (Cronbach’s alpha for the child’s self-reports = 0.901; parents’ proxy reports = 0.847).

### 2.2. Parental PTG

For the assessment of the parent’s own level of PTG (parent self-reported PTG), we used the Posttraumatic Growth Inventory (PTGI) [29]. The PTGI is the most widely used measure for assessing the positive changes following a traumatic experience in adults. It consists of 21 items that cover PTG in the domains of relating to others, new possibilities, personal strength, spiritual change and appreciation of life. For example, parents are asked if they “*have greater sense of closeness with others*” or “*have a greater feeling of self-reliance*”. The reliability of the PTGI was good (Cronbach’s alpha = 0.937).

For all the methods, the child’s illness was explicitly stated as the traumatic event to which all the items relate for both the survivors and the parents.

### 2.3. Analysis

As the first step, we performed a preliminary correlation analysis to assess mutual relationships of all the included PTG variables, followed by the analysis of the parent–child agreement on the level of child PTG at the individual level using intraclass correlation (two-way random, absolute agreement, single measures). For the analysis of the parent–child agreement at the group level, we used a paired sample *t*-test comparing the means of the self- and proxy-reported PTG scores. Inspired by the analysis reported by Clawson et al. [20] who used a similar approach for analyzing the influence of the parents’ own level of PTS on the concordance of the child’s self-report and the parents’ proxy reports of PTS following pediatric cancer survival, we divided the sample of parents in our study into terciles, resulting in three groups differing in the level of parental PTG (low, medium and high). Then, we performed intraclass correlation and a paired sample *t*-test to compare the means of the child’s self-report and the parents’ proxy reports of PTG for each group of parental PTG separately in order to analyze the parent–child agreement at various levels of parental PTG. Finally, a hierarchical regression analysis was conducted to examine the influence of child self-reported variables on the parents’ proxy reports of child PTG over and above the parent self-reported PTG when controlling for child gender, age and time off-treatment. All the analyses were performed using SPSS 27.

## 3. Results

The sample used for this study included 127 parent–child dyads. Sample characteristics for the childhood cancer survivors included in the sample can be found in Table 1. The sample was almost balanced in the proportion of males and females; most of the survivors were treated for extracranial solid tumors or leukemia, only a minority had CNS-related tumors. The majority of the survivors did not suffer from any late effects of treatment (evaluated by a physician according to the Common Terminology Criteria for Adverse Events v3.0). The parents included in this study were recruited from the parents accompanying the survivors for the follow-up visits at the hospital. Both the survivors and their parents were administered a more complex set of questionnaires focused on the quality of life and related concepts (including PTG). The sample in this study included only parent–child dyads, with complete data for parent- and child self-reported PTG as well as the parents’ proxy reports of PTG in the child. The survivors and their parents filled out a set of questionnaires separately in a paper–pencil form during a hospital visit. Of all the parents’ reports, 88 (69%) were provided by mothers, 9 (7%)—by fathers, 16 (13%)—by both parents together. For 14 (11%) parental reports, the information about the reporter (mother/father/both) is not available.

Table 2 presents the results of the correlation analysis of the child and parent self-reported PTG with the parent proxy-reported PTG in the child using the Pearson correlation coefficient. The parent–child agreement in child PTG was further analyzed using the intraclass correlation (ICC) of the child self-reported PTG and the parent proxy-reported PTG in the child to measure the level of absolute agreement in the two reports. Both the Pearson and ICC coefficients showed a poor level of the parent–child agreement (based on the differentiation proposed by Eiser and Morse [15] defining correlations below 0.3 as a poor level of agreement). The parents’ proxy report of child PTG was more closely tied to the parents’ own PTG than to the child self-reported PTG. The child self-reported PTG was further weakly positively related to the level of PTG.

A significant difference between the mean child self-reported PTG (M = 34.16, SD = 9.61) and the parents’ proxy reports of PTG in the child (M = 36.01, SD = 7.28) was found using the paired sample *t*-test for the whole sample (t_(126)_ = 2.04, *p* = 0.044). The parents reported higher levels of PTG in their child than the children themselves. To analyze the influence of the parents’ own level of PTG on the comparison of the mean child’s self-reports and the parents’ proxy reports of PTG, paired sample *t*-tests were performed in the bottom, middle and top terciles of the parental level of PTG separately. Table 3 shows descriptive statistics for all the PTG scores (parents’/child’s self-reports and parents’ proxy reports) for each tercile of parental PTG. Both the parents’ self and proxy PTG reports were significantly different among the terciles, while the child self-reported PTG was not.

**Figure 1:**
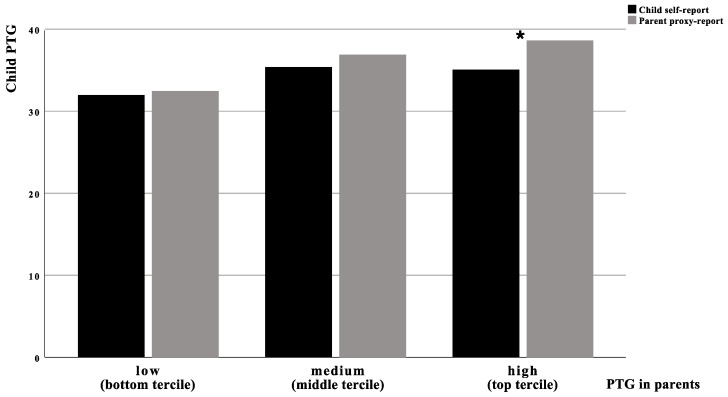
Level of child PTG according to self-reports and the parents’ proxy reports and the level of PTG in parents. * *p* ≤ 0.05.

Figure 1 presents the comparison of the child’s self-report and the parents’ proxy reports of PTG for individual terciles of the parents’ own PTG. At low and medium levels of parental PTG, paired sample *t*-tests yielded non-significant results. At the high level of parental PTG (top tercile), a paired sample *t*-test found a significant difference in the mean scores of the child self-reported PTG and the parents’ proxy reports of PTG in the child (t_(41)_ = 2.28, *p* = 0.028). The parents with a high level of PTG proxy-reported significantly higher levels of PTG in their child compared to the child self-reported PTG. The intraclass correlation of the child’s self-report and the parents’ proxy reports of PTG remained significant only in the bottom tercile of parental PTG (r = 0.40, *p* = 0.005).

Regression analysis was used to further analyze the influence of the parents’ own PTG on the parents’ proxy reports of PTG when controlling for child gender (0 = male, 1 = female), age at assessment and time off-treatment. A parents’ proxy report of child PTG was entered as the dependent variable, child gender, age and time off-treatment were entered in Step 1, parental PTG was entered in Step 2 and child self-reported PTG was entered in Step 3.

Table 4 shows the results of the regression analysis. After controlling for child gender, age and time off-treatment, the parents’ own PTG explained 21% of the variance in the parents’ proxy reports of child PTG. The addition of the child self-reported PTG significantly improved the level of variance explained by the model (ΔR2 = 0.041, *p* = 0.011). The final model explained 25% of the variance in the parents’ proxy reports of child PTG. Among the individual predictors in the final model, both the parents’ own PTG (*p* = 0.001) and the child self-reported PTG (*p* = 0.011) reached statistical significance, but the parents’ own PTG was a stronger predictor of the parents’ proxy reports than the child self-reported PTG.

## 4. Discussion

Psychosocial consequences of pediatric cancer include both positive (e.g., PTG) and negative (e.g., PTS) outcomes. Our understanding of the nature of PTG in childhood cancer survivors is a prerequisite for assessing its relationship with overall adaptation. To distinguish real PTG from positive illusions, some authors recommend corroboration of self-reported PTG by the reports of others (proxy reports). This study aimed to analyze (1) the level of concordance of parent- and child-reported child PTG, (2) the influence of the parents’ own PTG on the level of the parent–child concordance and (3) the influence of the parents’ and the child’s own PTG on the parents’ proxy report of PTG in the child.

Overall, the correlation analysis showed a poor level of the parent–child agreement on the level of child PTG and a positive association of the parents’ proxy reports of child PTG with the parents’ own PTG. A paired sample *t*-test for the whole sample revealed a significant difference between the child’s self-report and the parents’ proxy reports, with the parents reporting higher PTG scores than the children. However, a more detailed analysis of the parent–child agreement confirmed a significant difference in the mean PTG scores only for the subgroup including the parents with high PTG (top tercile), while the intraclass correlation remained significant only for the subgroup including the parents with low PTG (bottom tercile). Therefore, the parents’ proxy reports of child PTG were the most accurate at the lowest levels of the parents’ own PTG.

This result is in contrast with the results of Clawson et al. [20], whose analysis inspired our analytical plan. Their study focused on the parent–child concordance in PTS and found concordance for all the terciles of the parents’ PTS, with the highest correlation of the child’s self-report and the parents’ proxy reports of child PTS in the parents with the highest own level of PTS (top tercile). Our results for PTG show the opposite—better concordance in the parents with the lowest level of own PTG. As shown by the correlation and regression analyses performed in our study, the parents’ proxy reports of PTG in the child are more strongly associated with the parents’ own PTG than with the child self-reported PTG. Perhaps parents with higher PTG expect their children to perceive the more positive aspects of trauma or project their own feelings and perception of positive change following a trauma onto their child as proposed by Smith et al. [18] and Tomlinson et al. [17].

However, the differences between our result and the results reported by Clawson et al. [20] may also be related to the differences in the parent–child concordance for observable vs. non-observable domains described in the research on the quality of life. The parent–child concordance is known to be higher for more easily observable domains as compared to the less observable ones [15]. Following this distinction, child PTS characterized by symptoms of re-experiencing, avoidance, negative cognition and mood and increased arousal may be more easily observable for parents than child PTG. It may be easier for a child to communicate or express PTS, for example, as a fear or negative mood than to express a more abstract or complex nature of a positive change, for example, appreciation of life or personal strength. Thus, parents’ proxy reports of PTS in their child may be more accurate than parents’ proxy reports of PTG.

The research on the quality of life in childhood cancer survivors has also shown that parents often report poorer quality of life of their child than the children themselves [16]. In other words, parents may perceive pediatric cancer as more demanding, stressful or perhaps more traumatic than children—as an event with a more fundamental impact on the life of their child. If so, then it sounds plausible that they could also perceive a higher level of positive changes in the life of their child following cancer survival. Perhaps parents misestimate the impact of pediatric cancer on their child in both directions—towards the negative aspects (the parents report lower levels of the child’s quality of life) as well as the positive ones (the parents report higher levels of PTG in the child).

In terms of corroboration of self-reported PTG, using the reports of others has been recommended as a way to “verify” self-reports of PTG and discriminate actual positive changes from positive illusion, wishful thinking or similar concepts [22]. The idea behind this recommendation is that real positive changes should be noticeable by significant others, and a high degree of correlations of self-reports and proxy reports could serve to validate self-reports. The poor parent–child agreement found in our sample (ICC = 0.28) does not provide strong support for the idea of validation of the child self-reported PTG by the parents’ proxy report. However, given that the parents proxy-reported higher levels of PTG in the children than the children themselves, the poor parent–child agreement should not be understood as questioning the presence of PTG in children.

The literature on the corroboration of child PTG by parents is scarce. Only Yaskowich [8] analyzed the parent–child agreement on PTG following pediatric cancer survival. This study did not find a significant difference between the child’s self-report and the parents’ proxy report of child PTG. The majority of studies focused on the corroboration of self-reports and proxy reports of PTG in adults frequently reported a good level of agreement [23,24,26,30]. However, Helgeson [27] reported greater corroboration for negative aspects of breast cancer experience than for the positive ones. In this study, the mean scores of self-reports and proxy PTG reports were not significantly different but they were uncorrelated. The breast cancer survivors and their significant others reported the same level of PTG, but the couples’ reports of PTG in open-ended questions did not match each other. In our sample, we found a significant difference in the mean self- and proxy-reported scores with a significant but weak correlation indicating poor agreement. The parents’ proxy reports differ from the child’s self-reports at the group as well as individual level. As indicated by the results of the regression analysis, the parents’ proxy reports of PTG in the child are more strongly influenced by the parents’ own level of PTG than by the actual level of PTG in their child.

Based on these results, we suggest that when using the parents’ proxy reports about psychosocial outcomes in the child, the parents’ own adjustment should be taken into account (if applicable). The perspective of the parents may not match the perspective of their child. However, parents are valuable sources of information because children rely on their parents’ evaluation when accessing the healthcare system, as well as sources of psychosocial support.

Our study has several limitations. First, our sample of childhood cancer survivors included mainly adolescents (only 12 survivors (9.4%) were younger than 12 years at the time of assessment). Therefore, the generalizability of our results to younger survivors and their parents may be limited. Second, we used different measures of positive changes following pediatric cancer for the child’s (BFSC) and parents’ (PTGI) self-reports. Although benefit finding and posttraumatic growth can be understood as synonyms [31], some authors describe subtle differences between these two concepts [32]. Therefore, the weak parent–child correlation in self-reported PTG found in our study (0.18) may have been influenced by the methods used. Both the BFSC and PTGI are widely used methods for assessing the positive changes following a traumatic experience, but future studies should address the topic of the parent–child connection in PTG using corresponding methods. Third, the parent–child agreement may be influenced by several factors on the side of the parent including gender (mother vs. father) and sociodemographic characteristics of the parent (e.g., education, socioeconomic status). The method employed in our study does not include these variables. Future studies should address parent-child agreement and corroboration of child PTG by parents in more detail.

## 5. Conclusions

In sum, the results of this study showed poor parent–child agreement in PTG at both the individual and group levels. A good level of agreement was found only for the subgroup of parents with a low own level of PTG. The parents’ proxy reports of PTG in their child are more strongly connected to their own level of PTG than to the PTG reported by their child. The parents’ proxy reports of PTG in their child should be completed with the child’s self-reports whenever possible.

## Figures and Tables

**Table 1:** Sample characteristics of 127 childhood cancer survivors.

Variable	Characteristics	*N*	%	
Gender	Male	66	52.0	
	Female	61	48.0	
Diagnosis	CNS tumor	18	14.2	
	Leukemia	51	40.2	
	Extracranial solid tumor	58	45.7	
Late effects	No	61	48.0	
	Mild	36	28.3	
	Moderate	19	15.0	
	Severe	11	8.7	
		M	SD	Range
Age	Current age	15.89	3.24	11.5–25.3
Time	Time off-treatment (years)	8.15	2.41	3.3–17.8

CNS = central nervous system.

**Table 2:** Correlations of child and parent PTG with parent proxy reports of child PTG.

Variable	Child PTG	Parent PTG	Child PTG (Parent Proxy)	Child PTG (Parent Proxy)
Child PTG	—	0.18 *	0.29 **	0.28 **^,a^
Parent PTG		—	0.41 **	

* *p* ≤ 0.05; ** *p* ≤ 0.01; ^a^ intraclass correlation.

**Table 3:** Descriptive statistics for parental PTG terciles.

Tercile (N)	Parental PTG		Child PTG (Parent Proxy)		Child PTG	
	M (SD)	F	M (SD)	F	M (SD)	F
Bottom (42)	47.62 (16.21)	173.96 **	32.45 (7.53)	9.13 **	31.98 (9.13)	1.65
Middle (43)	75.47 (3.61)		36.91 (6.57)		35.40 (9.96)	
Top (42)	86.86 (4.67)		38.64 (6.40)		35.07 (9.56)	

** *p* ≤ 0.01.

**Table 4:** Results of the multiple regression analysis.

Variable	Child PTG (Parents’ Proxy Report)		
	B	SE B	β
Step 1 (R^2^ = 0.038)			
Age	0.380	0.205	0.169
Gender	−0.948	1.289	−0.065
Time off-treatment	−0.330	0.274	−0.109
Step 2 (R^2^ = 0.206 **; ΔR^2^ = 0.168 **)			
Age	0.379	0.187	0.169*
Gender	−0.813	1.176	−0.056
Time off-treatment	−0.321	0.250	−0.106
Parental PTG	0.155	0.031	0.410**
Step 3 (R^2^ = 0.247 **; ΔR^2^ = 0.041 *)			
Age	0.287	0.186	0.128
Gender	−1.297	1.165	−0.089
Time off-treatment	−0.287	0.245	−0.095
Parental PTG	0.140	0.030	0.370**
Child PTG	0.161	0.063	0.213*

* *p* ≤ 0.05; ** *p* ≤ 0.01.

## Data Availability

The data presented in this study are available in this article.

## References

[1] Taïeb O., Moro M.R., Baubet T., Revah-Lévy A., Flament M.F. (2003). Posttraumatic Stress Symptoms after Childhood Cancer. Eur. Child Adolesc. Psychiatry.

[2] Bruce M. (2006). A Systematic and Conceptual Review of Posttraumatic Stress in Childhood Cancer Survivors and Their Parents. Clin. Psychol. Rev..

[3] Kazak A.E., Alderfer M.A., Rourke M.T., Simms S., Streisand R., Grossman J.R. (2004). Posttraumatic Stress Disorder (PTSD) and Posttraumatic Stress Symptoms (PTSS) in Families of Adolescent Childhood Cancer Survivors. J. Pediatr. Psychol..

[4] Okado Y., Tillery R., Howard Sharp K., Long A.M., Phipps S. (2016). Effects of Time since Diagnosis on the Association between Parent and Child Distress in Families with Pediatric Cancer. Child. Health Care.

[5] Tedeschi R.G., Calhoun L.G. (2004). Posttraumatic Growth: Conceptual Foundations and Empirical Evidence. Psychol. Inq..

[6] Barakat L.P., Alderfer M.A., Kazak A.E. (2006). Posttraumatic Growth in Adolescent Survivors of Cancer and Their Mothers and Fathers. J. Pediatr. Psychol..

[7] Michel G., Taylor N., Absolom K., Eiser C. (2009). Benefit Finding in Survivors of Childhood Cancer and Their Parents: Further Empirical Support for the Benefit Finding Scale for Children. Child Care Health Dev..

[8] Yaskowich K.M. (2002). Posttraumatic Growth in Children and Adolescents with Cancer. Ph.D. Thesis.

[9] Sharp K.M.H., Willard V.W., Okado Y., Tillery R., Barnes S., Long A., Phipps S. (2015). Profiles of Connectedness: Processes of Resilience and Growth in Children with Cancer. J. Pediatr. Psychol..

[10] Sharp K.M.H., Willard V.W., Barnes S., Tillery R., Long A., Phipps S. (2016). Emotion Socialization in the Context of Childhood Cancer: Perceptions of Parental Support Promotes Posttraumatic Growth. J. Pediatr. Psychol..

[11] Koutná V., Jelínek M., Blatný M., Kepák T. (2017). Predictors of Posttraumatic Stress and Posttraumatic Growth in Childhood Cancer Survivors. Cancers.

[12] Koutná V., Blatný M. (2020). Socialization of Coping in Pediatric Oncology Settings: Theoretical Consideration on Parent–Child Connections in Posttraumatic Growth. Front. Psychol..

[13] Wakefield C.E., McLoone J., Butow P., Lenthen K., Cohn R.J. (2013). Support after the Completion of Cancer Treatment: Perspectives of Australian Adolescents and Their Families. Eur. J. Cancer Care.

[14] Varni J.W., Limbers C.A., Burwinkle T.M. (2007). How Young Can Children Reliably and Validly Self-Report Their Health-Related Quality of Life?: An Analysis of 8,591 Children across Age Subgroups with the PedsQL 4.0 Generic Core Scales. Health Qual. Life Outcomes.

[15] Eiser C., Morse R. (2001). Can Parents Rate Their Child’s Health-Related Quality of Life? Results of a Systematic Review. Qual. Life Res..

[16] Schulte F., Wurz A., Reynolds K., Strother D., Dewey D. (2016). Quality of Life in Survivors of Pediatric Cancer and Their Siblings: The Consensus Between Parent-Proxy and Self-Reports. Pediatr. Blood Cancer.

[17] Tomlinson D., Plenert E., Dadzie G., Loves R., Cook S., Schechter T., Dupuis L.L., Sung L. (2021). Reasons for Disagreement between Proxy-Report and Self-Report Rating of Symptoms in Children Receiving Cancer Therapies. Support. Care Cancer.

[18] Smith L.E., Weinman J., Yiend J., Rubin J. (2020). Psychosocial Factors Affecting Parental Report of Symptoms in Children: A Systematic Review. Psychosom. Med..

[19] Russell K.M.W., Hudson M., Long A., Phipps S. (2006). Assessment of Health-Related Quality of Life in Children with Cancer: Consistency and Agreement between Parent and Child Reports. Cancer.

[20] Clawson A.H., Jurbergs N., Lindwall J., Phipps S. (2013). Concordance of Parent Proxy Report and Child Self-Report of Posttraumatic Stress in Children with Cancer and Healthy Children: Influence of Parental Posttraumatic Stress. Psychooncology.

[21] Phipps S., Long A., Hudson M., Rai S.N. (2005). Symptoms of Post-Traumatic Stress in Children with Cancer and Their Parents: Effects of Informant and Time from Diagnosis. Pediatr. Blood Cancer.

[22] Park C.L., Lechner S.C., Calhoun L.G., Tedeschi R.G. (2006). Measurement Issues in Assessing Growth Following Stressful Life Experiences. Handbook of Posttraumatic Growth: Research & Practice.

[23] Shakespeare-Finch J., Enders T. (2008). Corroborating Evidence of Posttraumatic Growth. J. Trauma. Stress.

[24] Blackie L.E.R., Jayawickreme E., Helzer E.G., Forgeard M.J.C., Roepke A.M. (2015). Investigating the Veracity of Self-Perceived Posttraumatic Growth. Soc. Psychol. Pers. Sci..

[25] Weiss T. (2002). Posttraumatic Growth in Women with Breast Cancer and Their Husbands: An Intersubjective Validation Study. J. Psychosoc. Oncol..

[26] Moore A.M., Gamblin T.C., Geller D.A., Youssef M.N., Hoffman K.E., Gemmell L., Likumahuwa S.M., Bovbjerg D.H., Marsland A., Steel J.L. (2011). A Prospective Study of Posttraumatic Growth as Assessed by Self-Report and Family Caregiver in the Context of Advanced Cancer. Psychooncology.

[27] Helgeson V.S. (2010). Corroboration of Growth Following Breast Cancer: Ten Years Later. J. Soc. Clin. Psychol..

[28] Phipps S., Long A.M., Ogden J. (2007). Benefit Finding Scale for Children: Preliminary Findings from a Childhood Cancer Population. J. Pediatr. Psychol..

[29] Tedeschi R.G., Calhoun L.G. (1996). The Posttraumatic Growth Inventory: Measuring the Positive Legacy of Trauma. J. Trauma. Stress.

[30] Tallman B.A., Lohnberg J., Yamada T.H., Halfdanarson T.R., Altmaier E.M. (2014). Anticipating Posttraumatic Growth from Cancer: Patients and Collaterals Experiences. J. Psychosoc. Oncol..

[31] Helgeson V.S., Reynolds K.A., Tomich P.L. (2006). A Meta-Analytic Review of Benefit Finding and Growth. J. Consult. Clin. Psychol..

[32] Casellas-Grau A., Ochoa C., Ruini C. (2017). Psychological and Clinical Correlates of Posttraumatic Growth in Cancer: A Systematic and Critical Review. Psychooncology.

